# Effect of Coenzyme Q_10 _on ischemia and neuronal damage in an experimental traumatic brain-injury model in rats

**DOI:** 10.1186/1471-2202-12-75

**Published:** 2011-07-29

**Authors:** Murat Kalayci, Mufit M Unal, Sanser Gul, Serefden Acikgoz, Nilufer Kandemir, Volkan Hanci, Nurullah Edebali, Bektas Acikgoz

**Affiliations:** 1 Department of Neurosurgery, Faculty of Medicine, Zonguldak Karaelmas University, 67600, Kozlu, Zonguldak/TURKEY; 2 Department of Biochemistry, Faculty of Medicine, Zonguldak Karaelmas University, 67600, Kozlu, Zonguldak/TURKEY; 3 Deparment of Pathology, Faculty of Medicine, Zonguldak Karaelmas University, 67600, Kozlu, Zonguldak/TURKEY; 4 Department of Anesthesiology; Faculty of Medicine, Zonguldak Karaelmas University, 67600, Kozlu, Zonguldak/TURKEY

## Abstract

**Background:**

Head trauma is one of the most important clinical issues that not only can be fatal and disabling, requiring long-term treatment and care, but also can cause heavy financial burden. Formation or distribution of free oxygen radicals should be decreased to enable fixing of poor neurological outcomes and to prevent neuronal damage secondary to ischemia after trauma. Coenzyme Q_10 _(CoQ_10_), a component of the mitochondrial electron transport chain, is a strong antioxidant that plays a role in membrane stabilization. In this study, the role of CoQ_10 _in the treatment of head trauma is researched by analyzing the histopathological and biochemical effects of CoQ_10 _administered after experimental traumatic brain injury in rats. A traumatic brain-injury model was created in all rats. Trauma was inflicted on rats by the free fall of an object of 450 g weight from a height of 70 cm on the frontoparietal midline onto a metal disc fixed between the coronal and the lambdoid sutures after a midline incision was carried out.

**Results:**

In the biochemical tests, tissue malondialdehyde (MDA) levels were significantly higher in the traumatic brain-injury group compared to the sham group (*p *< 0.05). Administration of CoQ_10 _after trauma was shown to be protective because it significantly lowered the increased MDA levels (*p *< 0.05). Comparing the superoxide dismutase (SOD) levels of the four groups, trauma + CoQ_10 _group had SOD levels ranging between those of sham group and traumatic brain-injury group, and no statistically significant increase was detected. Histopathological results showed a statistically significant difference between the CoQ_10 _and the other trauma-subjected groups with reference to vascular congestion, neuronal loss, nuclear pyknosis, nuclear hyperchromasia, cytoplasmic eosinophilia, and axonal edema (*p *< 0.05).

**Conclusion:**

Neuronal degenerative findings and the secondary brain damage and ischemia caused by oxidative stress are decreased by CoQ_10 _use in rats with traumatic brain injury.

## 1 Background

Head trauma is one of the most important clinical issues that not only can be fatal and disabling, requiring long-term treatment and care, but also can cause heavy financial burden. It ranks fourth among all the causes of death [1]. Brain injury secondary to trauma consists of the primary damage formed by the direct effect of forces faced and the subsequent secondary damage formed by the sequential reactions of various metabolic events [2-7].

A huge part of the secondary brain damage is induced by lipid peroxidation caused by free oxygen radicals released due to impairment of the balance between the various antioxidant mechanisms occurring after brain trauma.

The formation or distribution of free oxygen radicals should be decreased to enable the fixing of poor neurological outcomes and to prevent neuronal damage secondary to ischemia after trauma [2-8]. It is assumed that the antioxidant defense system cannot completely neutralize the free oxygen radicals occurring in ischemic tissues, particularly after reperfusion [6]. Agents inhibiting free oxygen radicals have also been reported to improve poor neurological outcomes in the central nervous system after trauma or ischemia by their therapeutic effects [2-9].

Coenzyme Q_10 _(CoQ_10_), a component of the mitochondrial electron transport chain, is a strong antioxidant playing a role in membrane stabilization [10]. Experimental studies have found that CoQ_10 _is effective in the treatment of ischemia-reperfusion injury [11,12]. Various studies have also shown that CoQ_10 _inhibits lipid peroxidation and is as effective as alpha-tocopherol against free oxygen radicals; moreover, tissue levels of CoQ_9 _and CoQ_10_decrease after transient cerebral ischemia [11-13]. CoQ_10 _has also been reported to decrease brain lactate levels and lessen the diameter of ischemic lesions in animal models [14-16].

In this study, the role of CoQ_10 _in the treatment of head trauma was researched by analyzing the histopathological and biochemical effects of CoQ_10 _administered to rats after experimental traumatic brain injury.

## 2 Methods

### 2.1 Animals

The study was approved by the Ethics Committee of the School of Medicine, University of Karaelmas. A total of 28 Wistar albino adult male rats, weighing 350-400 g on average, were used in this study. This animal study was conducted in the Central Experimental and Clinical Research Laboratory, Faculty of Medicine, University of Karaelmas. All the rats were placed in a room maintained at 22-25°C, with appropriate humidity and a 12-hour light cycle, and were provided with sufficient fluids and feed.

### 2.2 Traumatic Brain Injury

A moderate brain-injury model, described by Marmarou et al. [17] and modified by Uçar et al. [18], was applied for head trauma to become a closed one and lead to reproducible brain injury. Anesthesia under spontaneous respiration was enabled with intraperitoneal administration of 50 mg/kg body weight ketamine hydrochloride (Ketalar, 50 mg/ml, 10 ml vial, Pfizer İlaçları Ltd., Istanbul) and 10 mg/kg body weight xylazine hydrochloride (Rompun, 2% solution, 50 cc vial, Bayer-Türk İlaç Ltd., Istanbul). The rats were placed in a prone position on the table. A midline incision was made on the head, and the coronal and lambdoid sutures were placed. A metallic disc of 10-mm diameter and 3-mm thickness was fixed to the cranium using bone wax between the two sutures and in the midline. Trauma was applied at the point where the metal disc was placed in the midline. A lead object weighing 450 g was allowed to fall freely from a height of 70 cm through a copper tube onto the metal disc over the skulls of the rats.

### 2.3 Experimental Groups

The rats were divided into four equal groups. For rats in the sham group, a scalp incision was made (G1). A traumatic brain injury was induced in the rats of the G2, G3, and G4 groups. Rats in the G3 group were administered 2 ml of 0.9% NaCl (physiologic saline) immediately after trauma and at the 24th hour by gavage. The rats in the G4 group were administered CoQ_10 _at a dose of 10 mg/kg immediately after trauma and at the 24th hour by gavage.

All the rats were anesthetized with the above-mentioned agents at the 48th hour after trauma, and their brains were extracted immediately without any damage. Samples of neural tissue were obtained by excising the left frontoparietal lobes from the boundary of the interhemispheric fissure and were subjected to biochemical analyses. The remaining parts of the brains were maintained in formaldehyde solution for pathological analyses.

### 2.4 Biochemical Procedures

#### Tissue malondialdehyde measurement

Tissue malondialdehyde (MDA) was measured by high-performance liquid chromatography using Immundiagnostik (Bansheim, Germany) kits on a 10% homogenate of the tissue in 50-mmol Tris-HCl buffer (pH 7.4). Sample separation was conducted with an analytical column (Phenomenex, USA). The mobile phase was injected into the system at a velocity of 1.0 ml/min with the help of an isocratic pump. Measurements were conducted using a fluorescence detector fitted in a Zivak Technologies apparatus under wavelengths of excitation and emission of 515 and 553 nm, respectively. The MDA concentrations were evaluated as nmol/g tissue divided by the tissue weight in grams.

#### Tissue superoxide dismutase activity measurement

Superoxide dismutase (SOD) was measured by the method of Sun et al. [19] using 10% tissue homogenate in Tris buffer. The method is based on the principle that the reduction of nitroblue tetrazolium (NBT) is inhibited by the xanthine-xanthine oxidase system, which acts as a superoxide generator. Inhibition percentage was calculated using the following formula:

Sample absorbances were measured in a Shimadzu UV-1601 spectrophotometer at 560 nm. One unit of SOD was defined as the amount of enzyme causing 50% inhibition in the rate of NBT reduction. SOD activity was expressed as units per milligram protein.

#### Paraoxonase measurement

Serum paraoxonase (PON) activities were measured as per the method of Eckerson et al. [20]. This method is based on the measurement of the rate of hydrolysis of paraoxon by the PON enzyme to form *p*-nitrophenol. Serum PON enzyme activity, in units of IU/L, was calculated by measuring the absorbance changes at 412 nm caused by the *p*-nitrophenol formed between the zero and the fifth minutes; the molar extinction coefficient of *p*-nitrophenol, 16.900 mol^-1 ^cm^-1^, was used for the calculations [20].

### 2.5 Histopathological Procedures

The brain parenchymal tissues of all rats in all the groups were embedded in paraffin and fixed with 10% buffered formalin for 24 hours. Using a microtome, 5- μm-thick serial sections were taken from the paraffin blocks and stained with hematoxylin and eosin (H&E) for routine histopathological observations. Sections of all tissue samples were observed under a light microscope (Leica, DMLS) in the Department of Pathology, Faculty of Medicine, Zonguldak Karaelmas University.

A semiquantitative scoring system, ranging between zero and three, was used for grading both the histopathological changes (vascular congestion, intraparenchymal hemorrhage, inflammation, neuronal loss, and gliosis) and the neuronal degenerative signs (nuclear pyknosis, nuclear hyperchromasia, cytoplasmic eosinophilia, and axonal edema) in the brain tissues of all histological samples. Nine different parameters assessed histopathologically were scored as follows: 0: absent; 1: mild; 2: moderate; and 3: dense/common.

### 2.6 Statistical Analysis

Data were evaluated in computerized media using the software SPSS 11.5. Results are presented as frequency, median, minimum, and maximum values. Kruskal-Wallis variance analysis was used for comparison of the biochemical values among groups. In case any difference was found, double-group comparisons were carried out according to Conover. Likelihood ratio and Fisher exact chi-square test were conducted for comparing the results of the histopathological assessment; *p *values less than 0.05 were considered significant.

## 3 Results

### 3.1 Biochemical findings

The biochemical results of the groups are presented in Table 1. The MDA levels were increased, with statistical significance (*p *< 0.05) in the G2 group compared with G1. In G4, to which CoQ_10 _was administered immediately after trauma, a statistically significant decrease (*p *< 0.05) in the MDA levels was noticed when compared to G2; moreover, this level was less than that in G1. The MDA level was additionally found to be higher in G3 than in G2, without any statistical significance. SOD levels were significantly lower in G2 and G3 than in G1 (*p *< 0.05). Comparing the SOD levels of the four groups G4 to G1, G4 had SOD levels ranging between those of G1 and G2, and no statistically significant increase was detected. In terms of the levels of the PON enzyme, no statistical difference was found between G2, G3, and G4 compared with G1.

**Table 1:** MDA, SOD and paraoxonase alterations among groups (median, minimum-maximum)

	G1		G2		G3		G4	
	**Median**	**min-max**	**median**	**min-max**	**median**	**min-max**	**median**	**min-max**

MDA	6.20 (4.54-8.71)		7.66 (6.34-10.12) *		8.40 (6.40-12.13) ‡		6.04 (4.79-6.96) #	
SOD	6.15 (4.49-6.98)		4.44 (3.44-6.15) *		5.25 (4.26-5.41) ‡		5.11 (4.61-6.29)	
PARAOXONASE	37.14 (19.10-51.10)		45.42 (17.74-53.47)		21.05 (18.92-82.80)		27.44 (15.14-64.35)	

*p > 0.05 (when compared to sham group).

#P < 0.05 (when compared to trauma group).

‡P < 0.05 (when compared to sham group).

### 3.2 Histopathological Findings

The parenchymal features of the white and grey matter, the neuronal morphology, and the vascular structures were assessed to be normal in the G1 samples. In the histopathological samples of G2 and G3, marked edema in the white and grey matter, in addition to vascular congestion were observed in the trauma-applied brain parenchyma tissue, along with neuronal injury findings, including hyperchromasia in neuronal nuclei, nuclear pyknosis, cytoplasmic eosinophilic degeneration, and axonal edema. Focal neuronal loss and gliotic zones were seen in areas consistent with the trauma site. Histopathological findings of the G2 and G3 groups were different from those of G1, with statistical significance in terms of parenchymal congestion, neuronal loss, gliosis, nuclear pyknosis, nuclear hyperchromasia, cytoplasmic eosinophilia, and axonal edema (*p *< 0.05). Again, no significant difference was observed between the sham group (G1) and the other three groups (G2, G3, and G4) with reference to intraparenchymal hemorrhage and inflammation.

**Figure 1:**
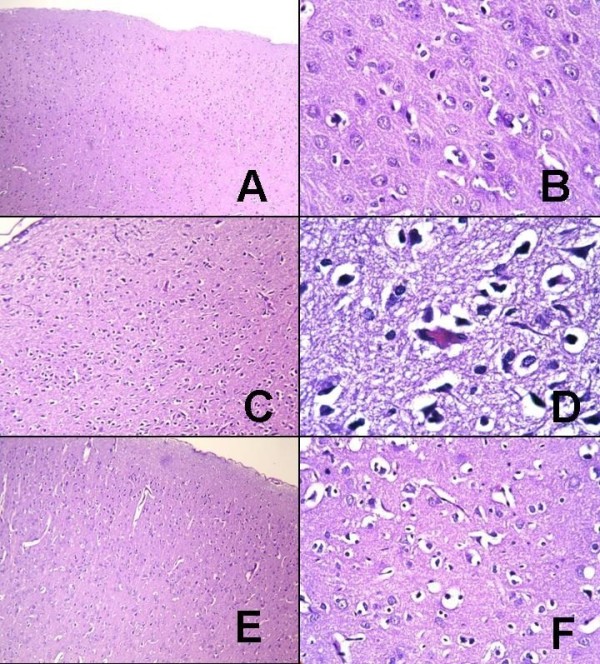
**Histological appearances of the groups**. A, B: G1; histological appearances of normal brain parenchyma (hematoxylin and eosin (H&E), A: × 100, B: × 400). C, D: G2; edema in brain parenchyma, pyknotic changes (green arrow), nuclear hyperchromasia, and cytoplasmic eosinophilia (red arrow) in neurons are observed (H&E, C: × 100; D: × 1000, oil immersion). E, F: G4; axonal edema and moderate reactive and degenerative changes in neuronal structures are seen (H&E, E: × 100; F: × 400).

When comparing the histopathological samples of G4 to those of G2 and G3, a statistically significant difference was detected in terms of vascular congestion, neuronal loss, nuclear pyknosis, nuclear hyperchromasia, cytoplasmic eosinophilia, and axonal edema (*p *< 0.05). Moreover, the intensity of the signs of neuronal degeneration in G4 was obviously less than those in G2 and G3. However, no statistically significant difference was observed among the three groups (G2, G3, and G4) in terms of gliosis, intraparenchymal hemorrhage, and inflammation (Table 2 and Figure 1).

**Table 2:** Parenchymal features and neuronal morphology distributions among groups

Histopathologycal Features	G1	G2	G3	G4	Total
Neuronal loss Score					

0	7	0	1	4	12

1	0	6	4	3	13

2	0	1	2	0	3

Total	7	7	7	7	28

Neuronal edema Score					

0	7	0	0	0	7

1	0	0	0	5	5

2	0	5	6	2	13

3	0	2	1	0	3

Total	7	7	7	7	28

Nuclear pyknosis Score					

0	7	0	0	0	7

1	0	0	1	5	6

2	0	5	6	2	13

3	0	2	0	0	2

Total	7	7	7	7	28

Neuronal hyperchromasia Score					

0	7	0	0	0	7

1	0	0	0	6	6

2	0	5	7	1	13

3	0	2	0	0	2

Total	7	7	7	7	28

Cytoplasmic eosinophilia Score					

0	7	0	0	1	8

1	0	2	3	6	11

2	0	5	4	0	9

Total	7	7	7	7	28

Parenchymal congestion Score					

0	7	0	0	0	7

1	0	3	5	6	14

2	0	4	2	1	7

Total	7	7	7	7	28

Parenchymal hemorrhage Score					

0	7	6	7	7	27

1	0	1	0	0	1

Total	7	7	7	7	28

Inflammation Score					

0	7	5	7	7	26

1	0	2	0	0	2

Total	7	7	7	7	28

Gliosis Score					

0	7	0	0	2	9

1	0	4	7	5	16

2	0	3	0	0	3

Total	7	7	7	7	28

## 4. Discussion

Head trauma may lead to primary and secondary damages in neural tissue. Secondary factors that have been shown to develop subsequent to traumatic brain injury are ischemia, cerebral edema, and free oxygen radical-based lipid peroxidation [2-7]. Lipid peroxidation leads to dysfunction of membrane because of changes in the lipid structure of the membrane. Secondary cell injury is observed subsequent to inflammation, edema, chemotaxis, and increased vascular permeability with the development of free oxygen radicals, influencing other components of the cell [21-23].

Antioxidants inhibit lipid peroxidation by preventing the peroxidative chain reaction and/or picking up the reactive-oxygen derivatives. Endogenous antioxidants include mitochondrial cytochrome oxidase, SOD, catalase, glutathione peroxidase, glutathione-S-transferase, hydroperoxidase, and CoQ. CoQ_10 _is the only fat-soluble antioxidant synthesized endogenously, and it is present in tissues in the active form (reduced) independently from the diet [24].

The method described by Marmarou et al. [17] and modified by Uçar et al. [18] was used as the trauma model in our study. We designed the model to analyze the protective effect of CoQ_10 _on neuronal damage. In the method of Marmarou et al. [17], the area between the coronal and lambdoid sutures was targeted; a diffuse brain injury was caused by allowing an object of 450 g to fall freely from an altitude of two meters through a plexiglass tube. A possible fracture of the calvarium was prevented by a stainless steel plate stuck over the calvarium, similar to the method of our application. In the study of Marmarou et al., of the 54 rats, mortality and cranium fracture were found in 44% and 12.5%, respectively. We applied the moderate head-trauma model described by Uçar et al. [18] because Marmarou et al.'s model has both high mortality and risk of posttraumatic seizure. Accordingly, we arranged for an object of 450 g to fall through a plexiglass tube from an altitude of 70 cm onto target points around the coronal suture. The total scheduled number of rats in the trauma groups was 21 in our study. Among these, four rats died of trauma, and therefore, four additional rats were exposed to trauma and included into the study. Hence, a total of 25 rats were subjected to trauma and four (16%) died, while no cranium fracture was detected in any rat.

CoQ_10_, whose use in many neurological, cardiac, oncological, and immunological diseases has been studied, has been shown to be protective against different forms of tissue damage [25-28]. However, the efficacy of CoQ_10 _in a moderate head-trauma model has not been analyzed before.

Administration of a 10 mg/kg dose immediately after the trauma, which we applied, has been reported to have a protective effect against ischemic injury in experimental models, such as cerebral ischemia, tissue ischemia-reperfusion damage, and spinal cord trauma [12,26,29-32]. Li et al. [33], in a different study, showed that the administration of CoQ_10 _three hours after focal and global ischemia, which they formed by vessel occlusion in rats, does not have a protective effect against neural injury [33].

Ostrowski [12] induced cerebral ischemia by administrating intraventricular endothelin to rats in his study. He showed that SOD activity increased significantly in samples obtained by killing the subjects after he injected 10 mg/kg CoQ_10 _intraperitoneally at the end of the 24th hour. His interpretation of this result was that CoQ_10 _would decrease cerebral ischemic injury if the endogenous SOD activity was higher [12].

Erol et al. [29] found in their ischemia-reperfusion model that MDA levels were significantly lower in the group in which they applied a single dose of 10 mg/kg CoQ_10 _intraperitoneally than that in control group. They concluded that this could be attributable to CoQ_10_, which had an antioxidant effect by lowering lipid peroxidation.

Kerimoğlu et al. [32] analyzed the efficacy of CoQ_10 _on experimental spinal cord injury induced by placing extradural aneurysm clips at the T4-5 level in rats, and they found that the SOD activity of only the trauma-applied group was significantly lower than that of the control group (*p *< 0.05). Although the MDA levels were less than those in the control group, the result was not considered statistically significant. Again, although edema was significantly more frequent in the control groups compared to others during histopathological assessment, no statistical difference was observed in their study between the methylprednisolone, CoQ_10_, and methylprednisolone + CoQ_10 _groups in terms of edema and hemorrhage.

Herein, we measured (1) the levels of MDA, one of the products of lipid peroxidation, for evaluating oxidative stress, and (2) the activities of SOD and PON, for analyzing the antioxidant capacity. A statistically significant reduction (*p *< 0.05) in the MDA levels was seen in the group administered CoQ_10 _immediately after trauma (G4) compared to the group to which only trauma was applied (G2), and even lower results than that in the sham group (G1) were detected. When evaluating the results of SOD, an endogenous antioxidant enzyme functioning as a member of the defense mechanism against oxidative stress, values in the G4 group were found to be similar to that of G1, although that did not reach any statistical significance. These findings suggest that CoQ_10 _is a neuronal protector against oxidative damage in traumatic brain injury and that it functions by inhibiting lipid peroxidation. When assessing the data of PON, an antioxidant enzyme associated with the high-density lipoprotein structure, no difference was detected among the different groups.

From the results of our histomorphological assessment, it has been concluded that in rats, administration of CoQ_10 _after traumatic brain injury (G4 group) reduces vascular congestion, neuronal loss, nuclear pyknosis, nuclear hyperchromasia, cytoplasmic, and axonal edema in a statistically significant manner when compared to the G2 and G3 groups (*p *< 0.05). Moreover, by administering CoQ_10_, neuronal degenerative findings were significantly less intense (*p *< 0.05).

## 5. Conclusions

The results of this study suggest that CoQ_10 _support immediately after brain injury prevents lipid peroxidation due to the antioxidant activity of CoQ_10_; CoQ_10 _administration is also effective in protecting against secondary neuronal damages after trauma. However, the main limitation of our study is that we investigated only the short-term effects of CoQ_10 _in traumatic brain injury. We cannot conclude whether CoQ_10 _causes any neuroprotection as a long-term effect. Therefore, more studies should be carried out to evaluate the short- and long-term effects of CoQ_10 _in secondary neuronal damages after trauma and to elucidate both its exact mechanism of action and clinical efficacy.

## Competing interests

The authors declare that they have no competing interests.

## Authors' contributions

MK conceived the study; collected and analyzed data; carried out *in vivo *experiments; interpreted data; and drafted the manuscript. MMÜ participated in the design of the study; collected and analyzed data; carried out *in vivo *experiments; interpreted data; and helped to draft the manuscript. ŞG participated in the design of the study; collected and analyzed data; carried out *in vivo *experiments; interpreted data; and helped to draft the manuscript. ŞA participated in the design of the study, carried out biochemical tests, and helped to draft the manuscript. NK participated in the design of the study, carried out histopathology tests, and helped to draft the manuscript. VH participated in the design of the study and helped to draft the manuscript. NE participated in the design of the study. BA participated in the design and coordination of the study. Moreover, all the authors have read and approved the final manuscript.
